# Poster Session I - A111 NURSE-LED TARGET-CONTROLLED INFUSION OF PROPOFOL FOR ENDOSCOPY: A PILOT STUDY

**DOI:** 10.1093/jcag/gwaf042.111

**Published:** 2026-02-13

**Authors:** C H Tsai, K Reti, F Forsyth, K Anderson, P J Belletrutti

**Affiliations:** Gastroenterology and Hepatology, University of Calgary, Calgary, AB, Canada; Foothills Medical Centre, Calgary, AB, Canada; Forzani & MacPhail Colon Cancer Screening Centre, Calgary, AB, Canada; University of Calgary, Department of Anesthesiology, Calgary, AB, Canada; Gastroenterology and Hepatology, University of Calgary, Calgary, AB, Canada

## Abstract

**Background:**

In Canada, endoscopies are typically performed with endoscopist-led, nurse-administered conscious sedation using fentanyl and midazolam. However, some patients cannot tolerate these procedures and require anesthetist-administered deeper sedation, an approach limited by resource constraints in most hospitals. Target-controlled infusion (TCI) of propofol allows precise, semi-automated titration of IV sedation.

**Aims:**

This study assessed the feasibility and effectiveness of a nurse-led TCI propofol–remifentanil protocol for patients who have had incomplete endoscopies under conscious sedation.

**Methods:**

From June 2024 to September 2025, 83 patients underwent endoscopy using a nurse-led TCI sedation protocol, including 64 had colonoscopies, 9 upper endoscopies, and 11 had combined procedures. The protocol, developed by the lead anesthetist and initially supervised by a senior anesthetist, began with remifentanil at 1.0 ng/mL, followed by propofol at 1.5 µg/mL once remifentanil reached 0.7 ng/mL effect site concentration. Thereafter, an anesthetist was nearby in the hospital and immediately available if needed. Medications were titrated every two minutes to achieve light to moderate sedation, defined as an Observer’s Assessment of Alertness and Sedation (OAAS) scale of 3–4. Time to sedation, total in-room time, safety outcomes, and clinician and patient satisfaction evaluated by qualitative questionnaires were recorded.

**Results:**

Mean time from remifentanil start to endoscope insertion was 11.5 ± 4.5 minutes for colonoscopy and 13.4 ± 5.6 minutes for EGD. In-room time was 53.2 ± 12.4 minutes for colonoscopy and 40.9 ± 9.4 for EGD. Most patients achieved the targeted OAAS score of 3–4; 4 colonoscopy patients reached deeper sedation with OAAS scale of 2. Two patients developed transient hypoxia managed with airway repositioning; one experienced hypotension treated with IV fluids; one required anesthesiology guidance for titration of propofol above the maximum protocol rate. Two patients presented to acute care within 30 days for minor, self-limited issues not related to sedation. Clinician and patient feedback indicated overall satisfaction as shown in Table 1.

**Conclusions:**

Nurse-led TCI propofol-remifentanil is a feasible, effective, and safe approach for patients requiring deeper sedation for difficult endoscopy. It may expand access to advanced sedation while maintaining safety under structured supervision; however, some patients may still benefit from general anesthesia. Individualized protocols and direct anesthetist oversight are essential during initial implementation.

**Funding Agencies:**

None

A111 Table 1: Clinician and patient satisfaction with sedation instrument (by number).

**Table:** 

		Very satisfied	Satisfied	Somewhat satisfied	Neither satisfied nor dissatisfied	Somewhat dissatisfied	Dissatisfied	Very dissatisfied
Clinician	Overall ease of the procedure	31	19	10	3	4	0	1
	Method of sedation compared with other methods of sedation	37	22	4	4	0	0	1
Patient	Overall satisfaction with the sedation experience	52	9	1	0	3	0	0
	Pain associated with the procedure	39	12	2	2	4	2	0

